# Establishing minimal clinically important differences and patient acceptable symptom state thresholds following birmingham hip resurfacing

**DOI:** 10.1007/s00402-024-05443-x

**Published:** 2024-07-09

**Authors:** Ignacio Pasqualini, Nickelas Huffman, Ahmed K. Emara, Alison K. Klika, John P. McLaughlin, Nathan Mesko, Peter J. Brooks, Nicolas S. Piuzzi

**Affiliations:** 1 Department of Orthopaedic Surgery, Orthopedic and Rheumatology Institute, Cleveland Clinic, 9500 Euclid Ave, A41, Cleveland, OH 44195 USA; 2 Department of Biomedical Engineering, Cleveland Clinic Foundation, 9500 Euclid Ave, Cleveland, OH USA

**Keywords:** Birmingham hip resurfacing, Minimal clinically important difference, Patient acceptable symptom state, Hip disability and osteoarthritis outcome score for pain, Hip disability and osteoarthritis outcome score physical function shortform

## Abstract

**Introduction:**

Birmingham Hip Resurfacing (BHR) has emerged as a compelling and innovative alternative to total hip arthroplasty (THA), especially among young, active patients. However, the Minimal Clinically Important Difference (MCID) and the Patient Acceptable Symptom State (PASS) thresholds have not yet been determined for patients undergoing BHR. Therefore, the current study aimed to (1) determine the MCID and PASS thresholds for both the Hip disability and Osteoarthritis Outcome Score (HOOS)-Pain and HOOS physical function shortform (PS), for patients who underwent BHR; and (2) identify factors influencing the achievement of MCID and PASS for HOOS-Pain and HOOS-PS.

**Methods:**

Prospectively collected data from patients undergoing BHR was analyzed. Patients with osteoarthritis and completed preoperative and 1-year postoperative PROMs were included. Distribution-based and anchored-based approaches were used to estimate MCID and PASS, respectively. The optimal cut-off point for PASS thresholds was calculated using the Youden index.

**Results:**

MCID for HOOS-Pain and PS were calculated to be 9.2 and 9.3, respectively. The PASS threshold for HOOS-Pain and PS were ≥ 77.7 and ≥ 87.3, respectively. The current study identified several factors affecting postoperative achievement of thresholds. Baseline Mental Component Summary (MCS) scores were a predictor for achieving MCID for postoperative HOOS-Pain, achieving MCID for postoperative HOOS-PS, achieving PASS for postoperative HOOS-Pain, and achieving PASS for postoperative HOOS-PS. Furthermore, baseline HOOS-Pain was a significant predictor for achieving MCID for postoperative HOOS-PS, achieving PASS for postoperative HOOS-Pain, and achieving PASS for postoperative HOOS-PS.

**Conclusions:**

MCID and PASS thresholds were established for HOOS-Pain and PS domains following BHR with most patients achieving these clinically meaningful benchmarks. Additionally, several factors affecting achievement of MCID and PASS were identified, including modifiable risk factors that may allow clinicians to implement optimization strategies and further improve outcomes.

## Introduction

Hip resurfacing has emerged as a compelling and innovative alternative procedure to total hip arthroplasty (THA) for the treatment of hip joint pathologies, especially among young, active patients [26]. The procedure involves preparing the femoral head with bone cutting tools and capping the remainder of the femoral head, similar to the way in which a dentist caps a tooth [27]. The femoral head can articulate with either the native acetabular cartilage or an implanted acetabular component [27]. The history and evolution of hip resurfacing has resulted in the creation of many different implant designs [1], with the BIRMINGHAM HIP Resurfacing System (BHR) (Smith & Nephew, Inc., Memphis, Tennessee) being one specific implant design. BHR only became available in the United States starting in 2006 [28], however, it was present and often practiced in many countries starting in the late 1990s [16]. As the indications for BHR in the United States have evolved over the past 20 years and currently limit the use of BHR to active males younger than 60 years old with a diagnosis of osteoarthritis and anatomy that allows the use of a femoral head component ≥ 48 mm [13, 43], it becomes increasingly vital to evaluate its outcomes and effectiveness comprehensively.

Patient-reported outcome measures (PROMs) play a crucial role in assessing patients’ subjective perceptions of their health status following surgery, allowing a deeper understanding of treatment effectiveness from patients’ perspectives [30]. The Hip disability and Osteoarthritis Outcome Score (HOOS) is one such specific PROM that comprehensively evaluates pain (HOOS-Pain) and functional ability (HOOS Physical function Shortform (PS)). BHR has been shown to provide excellent clinical and functional outcomes, comparable to THA in appropriately selected patients [12, 33]. In addition, BHR has demonstrated excellent implant survivorship up to 20 years [12], a finding unique to BHR as other hip resurfacing implants have historically shown high failure rates and poor survivability [14, 15, 25]. However, to fully comprehend the clinical significance of these PROMs, it is essential to determine specific thresholds that indicate clinically meaningful improvements post-arthroplasty [36]. These thresholds include the Minimal Clinically Important Difference (MCID) and the Patient Acceptable Symptom State (PASS).

The MCID denotes the minimum change in a PROM score between time points that patients perceive as beneficial or noteworthy, reflecting their personal assessment of improvement. On the other hand, the PASS represents the level of PROM score at which patients consider their clinical status to be satisfactory, providing a tangible measure of their postoperative well-being. While these values have been previously described for total hip and knee arthroplasty, they have not yet been determined for patients undergoing BHR. The need for establishing MCID and PASS thresholds for BHR lies in the fact that these thresholds may vary between different operative procedures. The studies that have previously established values for MCID and PASS are values specifically for THA patients [24, 37], and those values cannot be applied to any procedure other than THA. Similar studies have been performed for different knee procedures. Just as unicompartmental knee arthroplasty and total knee arthroplasty MCID and PASS thresholds differ [35], so may BHR from THA. Furthermore, the Therapeutic Goods Administration in Australia published information about a hazard alert and withdrew BHR from use in women starting in June 2015 [12, 19, 42], thus resulting in Smith & Nephew voluntarily withdrawing the use of BHR in women secondary to the significantly lower implant survivorship rates among women [12]. Therefore, patients undergoing BHR are men and often younger than the men and women undergoing THA. The different demographics of patients undergoing BHR compared to THA highlights another important reason for determining possible differences in MCID and PASS thresholds for patients undergoing different operative procedures.

Therefore, this study aimed to (1) determine the MCID and PASS threshold for the HOOS-Pain and HOOS-PS for patients who underwent BHR; and (2) identify factors influencing the achievement of MCID and PASS for HOOS-Pain and PS.

## Methods

### Study design and data sources

**Fig. 1:**
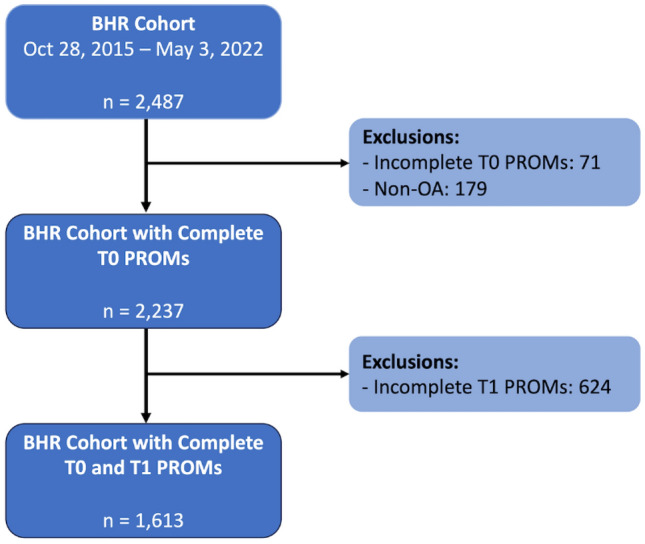
Strobe diagram depicting the inclusion and exclusion criteria of the current study

Data from a prospective study involving 2,487 men who underwent BHR at a single academic institution between October 28, 2015, and May 3, 2022, were analyzed. Patients without a diagnosis of osteoarthritis (n = 179, 7.20%), and those who did not complete baseline (n = 71, 2.85%) or one-year (n = 624, 27.9%) PROMs, were excluded from analysis (Fig. 1). Women were excluded from the study, as BHR was withdrawn from use in women starting in June 2015 [7, 12, 42]. Therefore, a total of 1613 patients completed one-year follow-up and were subsequently analyzed. Population demographics and baseline determinants are outlined in Table 1. All patients underwent BHR using a lateral surgical approach.

**Table 1:** Baseline demographic characteristics and 1-year outcomes of study population

Variable	Level	OA (N = 1613)	N completed data
Age (years) *		54 (8.8)	1613
Sex, N (%)	F	8 (0.5%)	1613
	M	1,605 (99.5%)	
BMI *		29.5 (5.5)	1613
Race, N (%)			1530
	White	1,409 (87.4%)	
	Black	103 (6.4%)	
	Other	18 (1.1%)	
ADI*		51.2 (24)	1541
CCI *		0.3 (0.8)	1613
Laterality, N (%)	L R	804 (49.8) 809 (50.1)	1613
Education (years)*		16 (2.8)	1613
Smoking, N (%)	Never	1,095 (67.8%)	1613
	Quit 6 m +	382 (23.7%)	
	Quit 0-6 m	41 (2.5%)	
	Current	95 (5.9%)	
Baseline PROMS			
	HOOS-Pain	42.5 (14.8)	1613
	HOOS PS	53.9 (16.8)	
1-year PROMS			1613
	HOOS-Pain	90.8 (14)	
	HOOS PS	91.9 (11.7)	
1-year Satisfaction, N (%)		1,469 (91.6)	1604

Results reported as Mean (Standard Deviation). *OA* osteoarthritis, *BMI* body mass index, *ADI* area deprivation index, *CCI* Charlson comorbidity index

The data was collected using our institutional database, a comprehensive and validated system-wide prospective data collection system that captures over 97% of all elective orthopaedic procedures within the healthcare system [5, 31, 39]. This database records essential information, including patient demographics, comorbidities, preoperative PROMs, socioeconomic factors, intraoperative details collected by the surgeon, postoperative 90-day healthcare utilization parameters, one-year reoperation and mortality rates, and PROMs [6, 10, 31, 32]. The joint-specific PROMs utilized in this investigation include the Mental Component Summary (MCS), HOOS-Pain, and HOOS-PS, which have been well-established and widely accepted in the evaluation of hip joint outcomes [8, 24, 30]. The study adhered to Institutional Review Board approval (IRB 06–196) and followed the guidelines set forth by Strengthening the Reporting of Observational Studies in Epidemiology (STROBE) to ensure rigorous and transparent reporting of observational studies. All patients provided written informed consent for the use of their data.

### Cohort stratification and outcome measures

PROMs were collected both preoperatively and one-year postoperatively. The primary outcome of this study was the extent of change in measured PROMs at one-year post-BHR compared to the preoperative baseline. Based on the extent of change in PROMs, thresholds for MCID in the aforementioned PROMs were determined. These thresholds were calculated using distribution-based methods, which involves calculating one-half of the standard deviation of the difference between pre- and postoperative PROM scores [21, 24]. Furthermore, PASS thresholds and corresponding percentages of patients achieving those thresholds at one-year postoperatively for each PROM were calculated.

### Data analysis

Means ± standard deviations (SD) of continuous variables were compared using independent sample *t*-tests, while categorical variable counts (%) were compared using *Chi*-squared tests.

The MCID was estimated to reflect the minimum PROMs improvement that translates into a patient-perceived change in their health status. MCID was calculated through distribution-based methods [4, 17, 37]. PASS values represented thresholds that indicated optimal patient satisfaction and were estimated using an anchor-based approach, which corresponds to a response to the following question at one-year: “Taking into account all the activities you have during your daily life, your level of pain, and also your activity limitations and participation restrictions, do you consider the current state of your hip satisfactory?” [22]. The percentage of patients who achieved PASS and MCID thresholds were also calculated. To determine cut-off values for each PROM, the Youden index [45] was calculated with its respective sensitivity, specificity, and area under the curve (AUC) using Receiver Operating Curve (ROC) analysis to best discriminate patient satisfaction.

A univariate analysis was performed for each potential predictor of MCID and PASS (age, race, Area Deprivation Index (ADI), Charlson Comorbidity Index (CCI), Body Mass Index (BMI), smoking, and baseline PROMs). A multivariate regression model was then performed to determine factors associated with the achievement of MCID and PASS. The odds ratio (OR) was then calculated for each variable based on the multivariate analysis. The statistical analysis was performed using Stata version 17 (Stata Corp). *P*-values < 0.05 were considered statistically significant.

## Results

### MCID and PASS thresholds

**Fig. 2:**
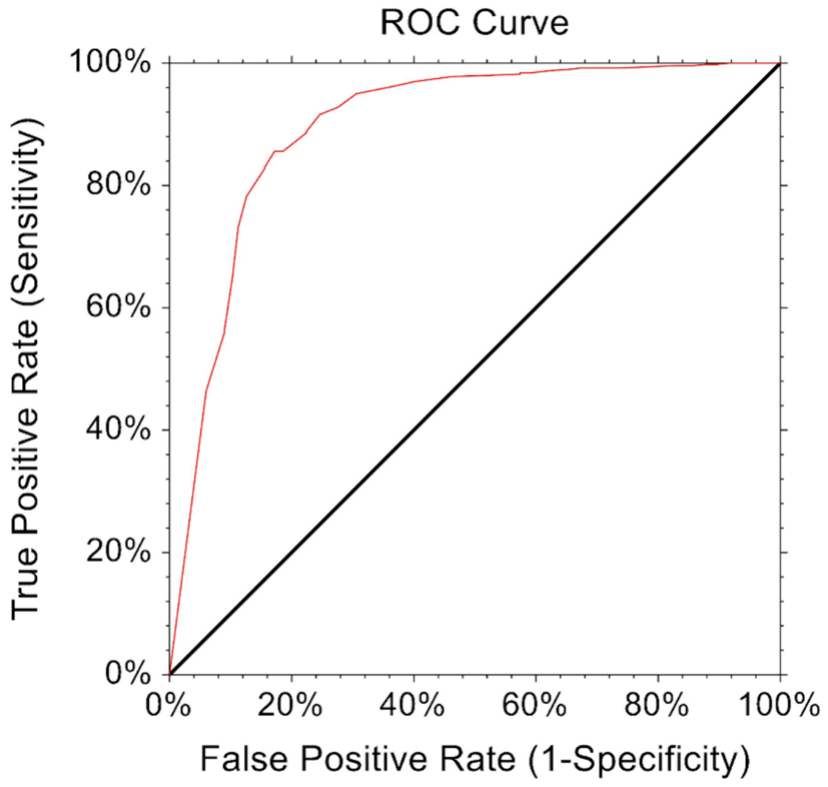
Receiver Operating Curve (ROC) for Hip disability and Osteoarthritis Outcome Score for pain (HOOS-Pain)

**Fig. 3:**
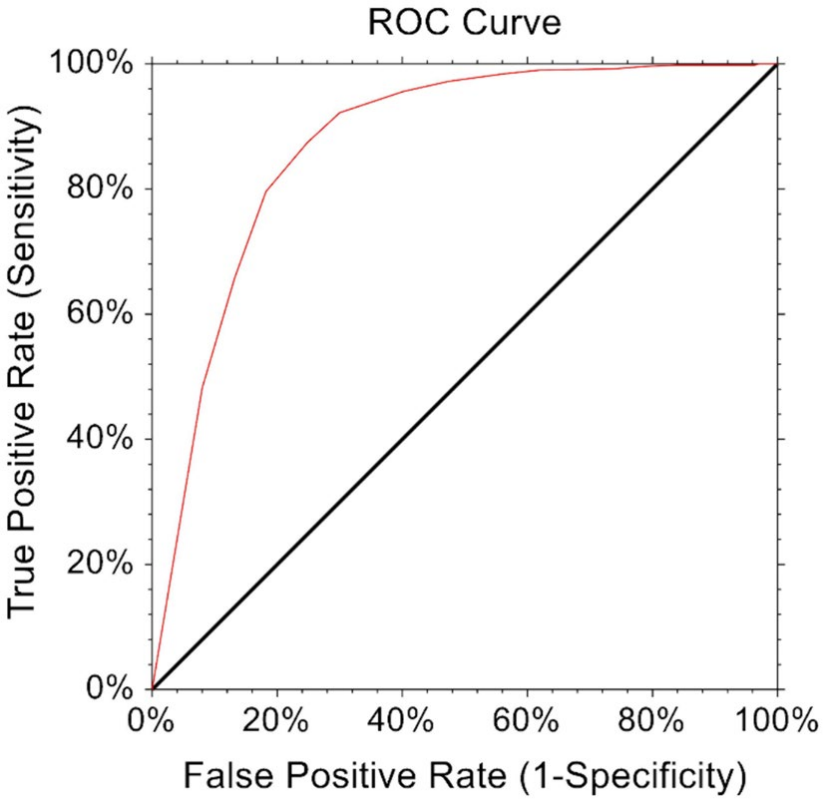
Receiver Operating Curve (ROC) for Hip disability and Osteoarthritis Outcome Score for the physical function shortform (HOOS-PS)

The MCID for HOOS-Pain was 9.2, with > 95% of patients achieving this MCID. For HOOS-Pain, the PASS threshold was ≥ 77.7, which was reached by > 85% of the cohort. Sensitivity, specificity, and AUC based on the ROC for HOOS-Pain are outlined in Table 2 and Fig. 2. The MCID for HOOS-PS was 9.3, with nearly 90% of patients achieving this MCID. The PASS threshold was ≥ 87.3, which was reached by nearly 80% of the cohort. Sensitivity, specificity, and AUC based on the ROC for HOOS-PS are outlined in Table 2 and Fig. 3.

**Table 2:** MCID and PASS thresholds for patients undergoing BHR

	MCID	% MCID	PASS threshold	% achieved PASS	Youden Index	Sensitivity	Specificity	AUC
HOOS-Pain	9.2	97.4%	≥ 77.7	85.8%	0.67	0.91	0.82	0.89
HOOS-PS	9.3	89.8%	≥ 87.3	78.6%	0.64	0.86	0.77	0.88

*AUC*  area under the curve

### Factors associated with achievement of MCID and PASS

Our analysis of the multivariate model reveals that baseline HOOS-Pain was a significant predictor for achieving MCID for postoperative HOOS-Pain. Baseline MCS scores were also significantly associated with achieving MCID for postoperative HOOS-Pain. However, baseline HOOS-PS scores were not associated with achieving MCID for postoperative HOOS-Pain (Table 3). In terms of achieving MCID for HOOS-PS, baseline HOOS-Pain scores were associated with achieving MCID for postoperative HOOS-PS, baseline HOOS-PS scores were strongly predictive of MCID postoperative HOOS-PS, and baseline MCS scores were associated with achieving MCID postoperative HOOS-PS (Table 3).

**Table 3:** Logistic regression of variables associated with achieving MCID for HOOS-Pain and HOOS-PS

Variable	Univariate analysis (*P*-value)	Multivariate analysis (*P*-value)	OR (95% CI)
*HOOS-Pain*			
Age	0.400	0.100	0.9 (0.9–1)
BMI	0.664	0.997	0.9 (0.9–1)
*Race*, *N* (%)			
White (ref)			
Black	0.861	0.738	1.2 (0.3–4.4)
*Other*			
ADI	0.036	0.077	0.9 (0.9–1)
CCI	0.485	0.676	0.9 (0.6–1.2)
*Smoking*, *N* (%)			
Never (ref)			
Quit 6 m +	0.005	0.101	0.3 (0–1.2)
Quit 0-6 m	0.448	0.329	0.6 (0.3–1.4)
Current	0.546	0.860	1.1 (0.2–5.4)
Baseline HOOS-Pain	< 0.001	< 0.001	0.9 (0.8–0.9)
Baseline HOOS-PS	0.150	0.073	1.03 (0.9–1)
Baseline MCS	< 0.001	< 0.001	1.08 (1.05–1.12)
*HOOS-PS*			
Age	0.934	0.799	0.9 (0.9–1.02)
BMI	0.502	0.160	0.9 (0.92–1.01)
*Race*, *N* (%)			
White (ref)			
Black	0.536	0.687	0.8 (0.3–2.1)
Other	0.536	0.756	1.4 (0.1–12)
ADI	0.876	0.369	0.9 (0.9–1)
CCI	0.304	0.329	0.8 (0.6–1.1)
*Smoking*, *N* (%)			
Never (Ref)			
Quit 6 m +	0.287	0.526	1.9 (0.2–15)
Quit 0–6 m	0.368	0.532	0.8 (0.4–1.4)
Current	0.521	0.556	1.4 (0.4–4.3)
Baseline HOOS-Pain	< 0.001	0.033	1.02 (1–1.04)
Baseline HOOS-PS	< 0.001	< 0.001	0.8 (0.86–0.89)
Baseline MCS	0.061	< 0.001	1.05 (1.03–1.08)

*MCID* minimal clinically important difference, *HOOS* hip disability and osteoarthritis outcome score, *PS* physical function shortform, *OR* odds ratio (calculated based on multivariate analysis), *CI* confidence interval, *BMI* body mass index, *ADI* area deprivation index, *CCI* Charlson comorbidity index, *MCS* mental component summary

When we shifted the focus to patients achieving PASS thresholds, a similar pattern emerged. Baseline HOOS-Pain and MCS both strongly predicted achieving PASS for postoperative HOOS-Pain. However, baseline HOOS-PS did not predict achieving PASS for postoperative HOOS-Pain (Table 4). Baseline HOOS-Pain and MCS also both predicted achieving PASS for postoperative HOOS-PS. However, baseline HOOS-PS was not a predictor of achieving PASS for postoperative HOOS-PS (Table 4). Interestingly, there were no baseline demographic characteristics associated with achieving MCID for either HOOS-Pain or HOOS-PS (Table 3). However, age and being a non-smoker were found to be significant predictors in achieving PASS for HOOS-Pain, and ADI was a significant predictor for achieving PASS for HOOS-PS (Table 4). Other patient characteristics, such as BMI, race, and CCI did not have significant impacts in our model.

**Table 4:** Logistic regression of variables associated with achieving PASS for HOOS-Pain and HOOS-PS

Variable	Univariate analysis (*P*-value)	Multivariate Analysis (*P*-value)	OR (95% CI)
*HOOS-Pain*			
Age	0.167	0.018	0.9 (0.95–0.99)
BMI	0.160	0.786	0.9 (0.9–1)
*Race*, *N* (%)			
White (ref)			
Black	0.143	0.663	0.8 (0.5–1.5)
Other	0.319	0.292	3 (0.3–23)
ADI	< 0.001	0.071	0.9 (0.9–1)
CCI	0.143	0.691	1.03 (0.8–1.2)
*Smoking*, *N* (%)			
Never (ref)			
Quit 6 m +	0.130	0.535	0.7 (0.3–1.8)
Quit 0-6 m	0.001	0.063	0.7 (0.5 – 1.01)
Current	0.001	0.049	0.5 (0.3–0.9)
Baseline HOOS-Pain	< 0.001	0.002	1.02 (1–1.04)
Baseline HOOS-PS	< 0.001	0.373	0.9 (0.9–1)
Baseline MCS	< 0.001	< 0.001	1.03 (1.02–1.05)
*HOOS-PS*			
Age	0.525	0.264	0.9 (0.9–1)
BMI	0.819	0.181	1 (0.9–1.04)
*Race*, *N* (%)			
White (ref)			
Black	0.094	0.545	0.8 (0.5–1.4)
Other	0.188	0.197	3.8 (0.4–30)
ADI	< 0.001	0.035	0.9 (0.98–0.99)
CCI	0.053	0.400	0.9 (0.8–1)
*Smoking*, *N* (%)			
Never (Ref)			
Quit 6 m +	0.007	0.091	0.5 (0.2–1)
Quit 0-6 m	0.015	0.281	0.8 (0.6–1)
Current	0.009	0.220	0.7 (0.4–1)
Baseline HOOS-Pain	< 0.001	0.015	1.01 (1–1.03)
Baseline HOOS-PS	< 0.001	0.151	1 (0.9–1.02)
Baseline MCS	0.061	0.002	1.01 (1–1.03)

*PASS* patient acceptable symptom state, *HOOS* hip disability and osteoarthritis outcome score, *PS* physical function shortform, *OR* odds ratio (calculated based on multivariate analysis), *CI* confidence interval, *BMI* body mass index, *ADI* area deprivation index, *CCI* Charlson comorbidity index, *MCS* mental component summary

## Discussion

This study established meaningful benchmarks for evaluating clinical success after BHR using PROMs. Specifically, we defined procedure-specific MCID and PASS thresholds for HOOS-Pain and physical function domains. Our findings demonstrated that the majority of BHR patients achieved these clinically significant HOOS benchmarks at 1-year postoperatively. These quantifiable MCID and PASS targets provide a framework for assessing meaningful improvements in patient health status and satisfaction after BHR. Additionally, our multivariate analysis identified preoperative HOOS-Pain, HOOS-PS, and MCS as primary drivers of achieving MCID and PASS thresholds following BHR. Overall, this study establishes valuable BHR-specific PROMs benchmarks to help define clinical success and identify high-risk patients preoperatively, thus guiding surgical decision-making and setting patient expectations.

Numerous studies have reported significant improvements in PROMs after BHR [2, 9, 23, 34]. For instance, Back et al. examined 230 BHR patients and observed significant improvements in Oxford Hip Scores (OHS), and Short Form (SF)-12 physical and mental component scores, with evaluations spanning at least two years after the procedure [2]. In another rigorous randomized trial, Costa et al. demonstrated significant improvements in OHS and health related quality of life assessed using the EuroQol (EQ-5D) over a five-year period in a cohort of 60 BHR patients [9]. Furthermore, Pascual-Garrido et al. conducted a study involving 79 BHR patients and reported improvements in modified Harris Hip Scores (mHHS), Western Ontario and McMaster Universities Arthritis Index (WOMAC) scores, SF-36 scores, and UCLA activity scores, evaluated at a minimum of five years following the surgery [34]. Similarly, we found that HOOS-Pain and HOOS-PS scores increased from preoperative to 1-year postoperative evaluation.

When comparing BHR to THA, Siqueira et al. performed an age-matched prospective cohort study of 707 BHR and 707 THA patients. At 1-year postoperative, they found no statistically significant differences in HOOS-Pain (median difference 1.4 points, p = 0.129) or HOOS-PS scores (median difference 2 points, p = 0.03) [41]. Overall, the collective findings from these PROMs evaluations consistently demonstrate significant improvements following BHR procedures, with pain and function outcomes closely matching those of THA.

The concept of MCID and PASS has been extensively investigated in the context of THA, with studies reporting patients reaching MCID and PASS in pain, functionality, and hip-related quality of life after THA [24, 37]. Notably, Lyman et al. [24] conducted a retrospective review in 2018 of 2,323 patients from a THA joint registry to determine the thresholds for MCID. The study utilized distribution-based methods and revealed MCID values of 9 points for HOOS-Pain and 7 points for HOOS-Joint Replacement [24]. Similarly, in a prospective analysis conducted in 2014 by Paulsen et al. [37], patients who underwent THA were examined to establish one-year patient PASS cut-points. The study identified PASS cut-points of 91, 88, and 83 for HOOS-Pain, PS, and hip-related Quality of Life (QoL), respectively. Notably, this study also uniquely analyzed PASS cut-points based on preoperative diagnosis, distinguishing between HOOS-Pain at 92 for idiopathic osteoarthritis versus 90 for other diagnoses, HOOS-PS at 88 for idiopathic osteoarthritis versus 86 for other diagnoses, and HOOS QoL at 84 for idiopathic osteoarthritis versus 77 for other diagnoses [37]. However, MCID and PASS values for BHR have not been fully described yet.

Harrison-Brown et al. examined changes in PROMs between 2 years and medium-term follow-up (median 9–10 years) after BHR. They found that patients may experience clinically meaningful decline in PROMs over time. However, the vast majority maintain scores within a satisfactory symptom state up to 10 years postoperatively [20]. They found that 18% of patients reported reductions in mHHS exceeding the MCID of 7.9 points. For WOMAC function, 21% had decreases surpassing the MCID of 9 points. However, nearly all patients remained above the PASS thresholds, which were 74 points for mHHS and 69.1 points for WOMAC function. Given the variability observed in MCID and PASS values across different joint arthroplasty populations, it is crucial to have specific and well-defined thresholds for different PROMs for each procedure, including BHR [11, 36]. Having such tailored metrics will enable more accurate assessments of patient outcomes and help healthcare professionals make informed decisions to optimize postoperative care and enhance patient satisfaction and quality of life. With our defined MCID threshold for pain calculated to be 9.2, the studied patient population achieved excellent outcomes, with > 97% of patients achieving MCID at postoperative 1-year follow-up. Similarly, with the MCID threshold for functional ability of 9.3, patients again had excellent outcomes with nearly 90% of patients achieving MCID at 1-year. When examining the PASS for pain threshold of ≥ 77.7, over 85% of patients reached satisfaction with their pain level postoperatively. Similarly, the PASS for functional ability threshold of ≥ 87.3 resulted in nearly 80% of patients reaching satisfaction with their postoperative functional ability. These results are similar to the percentage of patients achieving PASS for mHHS in the Harris-Brown study [3]. Furthermore, these results provide insight into the promising PROM results in patients undergoing BHR.

There are several well-known factors associated with worse postoperative PROMs in BHR patients, including female sex, low bone mineral density, higher BMI, presence of comorbidities, smoking, and femoral head diameter < 48 cm [20]. In addition, Harrison-Brown et al. elucidated several other early postoperative PROMs associated with patient dissatisfaction at later follow-up. Specifically, at 2-year follow up, PROM results showing Veterans RAND (VR)-36 Physical Component Score < 51, mHHS < 84, prior bone graft use, and counterintuitively, VR-36 Mental Component Score > 55 all predicted decreased patient satisfaction at later follow-ups [20]. Our analysis identified several preoperative factors that aligned with achieving meaningful improvement in HOOS-Pain and physical function after BHR. Worse pain, reflected by lower baseline scores for HOOS-Pain, was strongly associated with increased likelihood of reaching MCID and PASS benchmarks, likely because greater room for improvement exists when starting at greater baseline symptom severity [44]. Better preoperative mental health, reflected in higher MCS scores, also positively predicted attaining pain and function thresholds. This aligns with a previous study that analyzed THA patients and found that patients with worse preoperative mental health were more likely to be dissatisfied at 1 year postoperatively [29]. Additionally, results of the current study showed that factors such as age and being a non-smoker were significant predictors of achieving PASS for HOOS-Pain, and ADI was a significant predictor for achieving PASS for HOOS-PS. This is supported by the literature as prior studies analyzing THA patients have found that demographic variables such as younger age [38], smoking history [40], and ADI [18] are associated with postoperative outcomes. Taken together, these patient factors allow clinicians to appropriately set expectations for clinically significant gains in HOOS-Pain and physical function after BHR and provide a predictive framework for identifying patients who are likely to experience successful outcomes based on meaningful HOOS improvement.

Limitations of the current study include the fact that PROMs such as HOOS-Pain, HOOS-PS, and MCS may be limited by recall and expectancy bias, potentially influencing the postoperative results that patients report. Also, due to the limited number of patients who did not achieve MCID or PASS, the predictive power of the regression analysis results found in Tables 3 and 4 is limited. Postoperative complications such as conversion to THA, failure of BHR, periprosthetic joint infection, and implant survivorship were not included in the current study but are important considerations when analyzing postoperative outcomes. Future studies should analyze survivorship, postoperative complications, and adverse outcomes following BHR. In addition, we utilized the reliable and validated question from Kvien et al. [22] to determine PASS threshold from BHR. Our PASS threshold differed from the Paulsen et al. [37] study where a different PASS question was used to determine the PASS threshold for THA. This highlights the fact that several factors may affect the findings of different PASS thresholds, and future studies should continue to validate or refute findings of MCID and PASS thresholds. With regards to comparing BHR and THA indications, it is important to note that there have been many enhancements to THA implant bearing surfaces since the completion of the THA MCID and PASS PROM studies conducted by Paulsen et al. [37] in 2014 and Lyman et al. [24] in 2018, thus resulting in potential differences between THA and BHR outcomes today. Finally, using either distribution-based or anchor-based methods for determining MCID and PASS thresholds are both valid but can offer different results. The current study used distribution-based and anchored-based approaches to estimate MCID and PASS, respectively.

## Conclusion

Meaningful values for the MCID and PASS thresholds for both HOOS-Pain and PS domains were established for patients diagnosed with osteoarthritis who underwent BHR, providing valuable benchmarks for gauging the success of BHR from the patient’s perspective. Baseline factors associated with postoperative PROMs included a greater baseline MCS score and greater odds of achieving MCID and PASS in both postoperative pain and functionality,worse baseline pain and achievement of MCID in pain postoperatively, and less baseline pain and achievement of patient satisfaction in postoperative pain and functionality. Overall, the percentage of patients achieving MCID and PASS thresholds was high, underscoring the effectiveness of BHR in engendering perceptible improvements in patient-reported outcomes. Future work should aim to validate these thresholds in other populations and potentially incorporate them into shared decision-making tools to optimize patient outcomes following BHR.

## Ethics approval

The study adhered to Institutional Review Board approval (IRB 06–196) and followed the guidelines set forth by Strengthening the Reporting of Observational Studies in Epidemiology (STROBE) to ensure rigorous and transparent reporting of observational studies.

## Informed consent

All patients provided written informed consent for the use of their data.

## Data Availability

Data for the current study will not be deposited in a repository.
